# Laparoscopic versus robotic abdominal and pelvic surgery: a systematic review of randomised controlled trials

**DOI:** 10.1007/s00464-023-10275-8

**Published:** 2023-07-13

**Authors:** Michal Kawka, Yuman Fong, Tamara M. H. Gall

**Affiliations:** 1 Department of Medicine, Imperial College London, London, UK; 2 Department of Surgery, City of Hope Medical Center, Duarte, CA 91010 USA; 3 Department of HPB Surgery, The Mater Misericordiae Hospital, Dublin, Ireland

**Keywords:** Systematic review, Robotic surgery, Laparoscopic surgery, Surgical outcomes

## Abstract

**Background:**

The current evidence is inconclusive on whether robotic or laparoscopic surgery is the optimal platform for minimally invasive surgery. Existing comparisons techniques focus on short-term outcomes only, while potentially being confounded by a lack of standardisation in robotic procedures. There is a pertinent need for an up-to-date comparison between minimally invasive surgical techniques. We aimed to systematically review randomised controlled trials comparing robotic and laparoscopic techniques in major surgery.

**Methods:**

Embase, Medline and Cochrane Library were searched from their inception to 13th September 2022. Included studies were randomised controlled trials comparing robotic and laparoscopic techniques in abdominal and pelvic surgery. The study followed the Preferred Reporting Items for Systematic Reviews and Meta-Analyses (PRISMA) guidelines. Short-term, health-related quality of life, and long-term, outcomes were analysed.

**Results:**

Forty-five studies, across thirteen procedures, involving 7364 patients were included. All of the studies reported non-significant differences in mortality between robotic and laparoscopic surgery. In majority of studies, there was no significant difference in complication rate (*n* = 31/35, 85.6%), length of postoperative stay (*n* = 27/32, 84.4%), and conversion rate (*n* = 15/18, 83.3%). Laparoscopic surgery was associated with shorter operative time (*n* = 16/31, 51.6%) and lower total cost (*n* = 11/13, 84.6%). Twenty three studies reported on quality of life outcomes; majority (*n* = 14/23, 60.9%) found no significant differences.

**Conclusion:**

There were no significant differences between robotic surgery and laparoscopic surgery with regards to mortality and morbidity outcomes in the majority of studies. Robotic surgery was frequently associated with longer operative times and higher overall cost. Selected studies found potential benefits in post-operative recovery time, and patient-reported outcomes; however, these were not consistent across procedures and trials, with most studies being underpowered to detect differences in secondary outcomes. Future research should focus on assessing quality of life, and long-term outcomes to further elucidate where the robotic platform could lead to patient benefits, as the technology evolves.

**Supplementary Information:**

The online version contains supplementary material available at 10.1007/s00464-023-10275-8.

## Introduction

Minimally invasive surgery (MIS) is an established alternative to open surgery for several surgical procedures, providing unique benefits, including less postoperative pain, reduced morbidity, shortened hospital stay, and a quicker return to functional activity [1]. Laparoscopic surgery, is the most commonly utilised MIS platform, becoming the gold standard for common operations such as cholecystectomy, and appendicectomy [2, 3]. However, despite being first described in the early 1900s, laparoscopy was only widely adopted towards the end of the twentieth century [4]. The initial experience, with rudimental technology and lack of operative experience, was often associated with outcomes inferior to the gold-standard open procedures [5].

One of the most important advances in the field of MIS was the introduction of robotic platforms, which is often ascribed to Kwoh et al., who in the late 1980s described using a robotic system to assist in brain biopsies [6]. Since then, robotic operations have been performed in multiple surgical specialities, with the DaVinci surgical system being the most commonly used [7]. The appeal of robotic surgery over its open counterpart stems from the benefits shared with other MIS techniques. The 3D visual field and additional dexterity of movement provided by endowristed instruments as well as tremor elimination, allowing increased precision, gives the robotic platform a potential advantage over laparoscopy [8]. However, the additional high cost has stifled its widespread clinical adoption [9].

As such, comparisons of robotic and laparoscopic surgery have become a topic of interest in the surgical community. The current evidence base is inconclusive as to whether the advantages of robotic surgery result in improved patient outcomes to justify the increased cost to healthcare systems [10–12]. Systematic reviews may include cases with the early robotic experience of centres, potentially leading to inferior outcomes during the initial learning curve. Further, currently available reviews focus on short-term outcomes only, limiting the real-world implications of comparing surgical techniques [10–12]. As such, there is a need for an up-to-date synthesis of evidence, in light of advances in robotics and new platforms emerging on the market.

We aimed to systematically review all randomised controlled trials comparing robotic and laparoscopic techniques across major surgical procedures in general surgery, gynaecology and urology to compare short-term outcomes, patient-reported outcomes and long-term outcomes where possible.

## Methods

### Literature search

A systematic review was conducted to identify all prospective randomised clinical trials comparing robotic surgery with the laparoscopic approach, across major surgical procedures in urology, gynaecology, and general surgery (encompassing oesophagogastric surgery, hepato-pancreato-biliary surgery, and colorectal surgery). EMBASE, Medline, and Cochrane Register of Systematic Reviews were searched from inception to the 13th of September 2022. The systematic review was conducted according to the PRISMA guideline (see Supplementary Digital Content 1) [13]. The full search strategy is shown in Supplementary Digital Content 2. Papers meeting the inclusion criteria had their references and citations manually searched to identify papers potentially missed in the initial literature search.

### Screening and study selection

Records identified from the search were uploaded to Covidence for study selection. Following the removal of duplicate publications, the initial screening of studies was performed based on titles and abstracts by two independent investigators (MK, TMGH). In the event of disagreement between these reviewers, it was discussed with an independent reviewer to establish a consensus. The same process was adopted for full-paper screening.

### Eligibility criteria

Randomised, prospective studies directly comparing robotic surgery with laparoscopic surgery were included. If the study contained a third subgroup (e.g., open surgery), but a separate comparison between robotic and laparoscopic surgery was also made, the study was eligible for inclusion. Surgeries were included if they were performed within urology, gynaecology, and general surgery. Following the initial scoping search, surgeries were divided into four categories: (1) Complex Upper gastrointestinal Surgery, (2) Complex Lower Gastrointestinal Surgery, (3) Urological and Gynaecological Surgery, (4) Non-complex General Surgery. A full list of included procedures can be found in Supplementary Digital Content 2. Studies describing cardiac surgery, thoracic surgery, neurosurgery and orthopaedic surgery were deemed to be outside the scope of this review and therefore excluded. Studies not in English, describing paediatric (< 18 yo) populations, and studies not reporting on short-term outcomes, were excluded. Studies which did not stratify the outcomes between robotic surgery and a comparator group were also excluded.

### Data extraction

Once the studies for inclusion had been determined, data was extracted from each across three domains using a standardised data extraction instrument: metadata and context of the study, study characteristics, and outcomes data (including short-term outcomes, and where available, long-term outcomes, and quality of life outcomes). Short-term outcomes included 30-day or 90-day mortality (whichever one was used by the study), morbidity (including stratification into major and minor Clavien-Dindo complications where available), readmission rates, estimated blood loss, transfusion rates, conversion rates, the total length of stay (incl. intensive care unit stay where available). Operative time and total cost were also extracted. For the quality of life outcomes, the stratified results for each group were extracted, alongside information about the tool used to measure the quality of life. For long-term outcomes, overall survival and progression-free survival as well as each interval listed in the study were extracted.

### Risk of bias assessment

Risk of bias assessment of the studies included was conducted using the RoB-2 tool and was performed by two independent reviewers [14].

### Statistical analysis

Following data extraction, data were pooled across procedures and qualitatively summarised. If the results of the same RCTs were utilised in more than one study, the satellite studies were included in the review, however, their results were excluded from analyses. No formal meta-analysis was conducted. This review was not formally registered; however, a pre-specified protocol was used for its conduction.

## Results

### Study characteristics

**Fig. 1:**
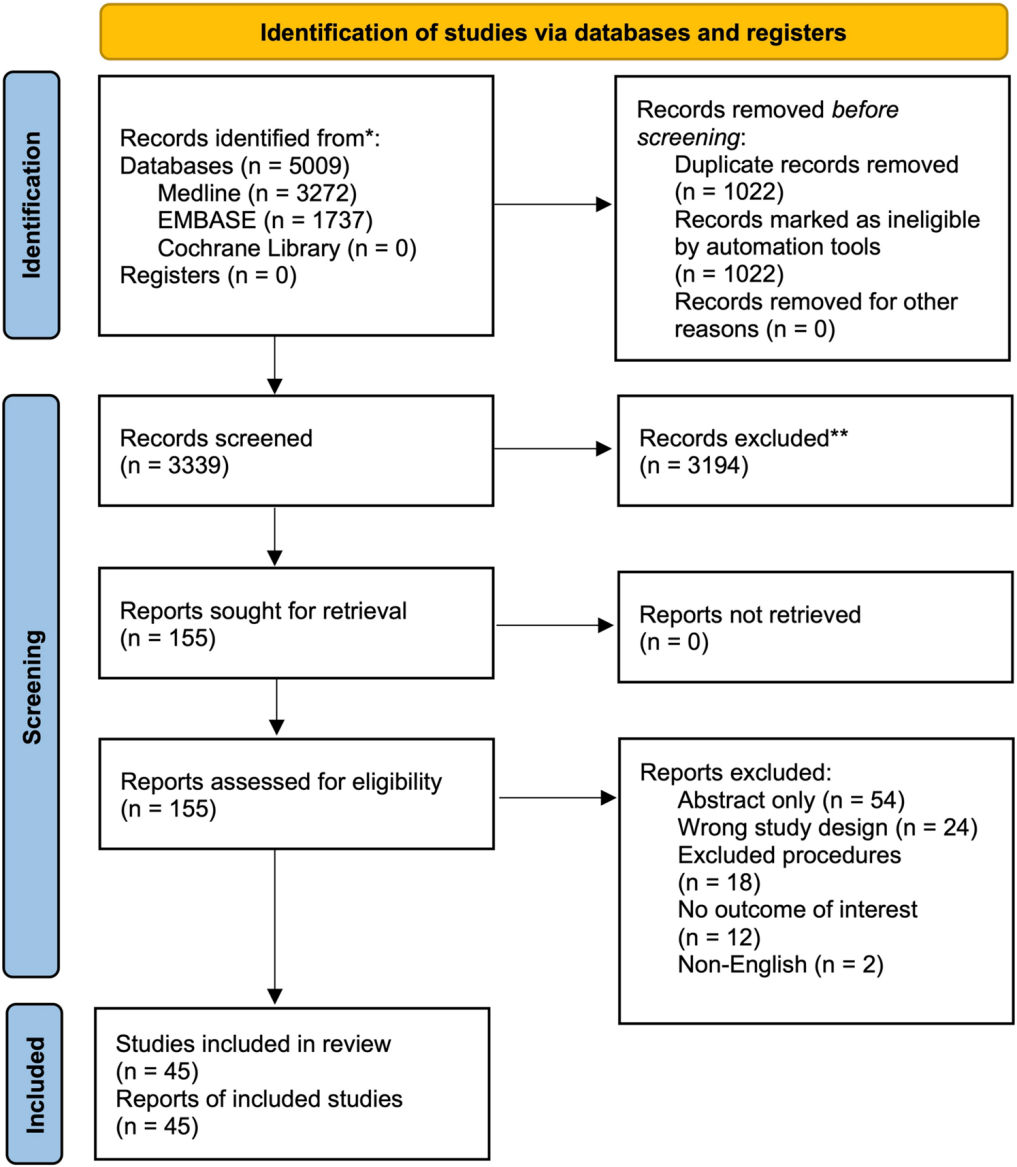
PRISMA Flowchart

**Fig. 2:**
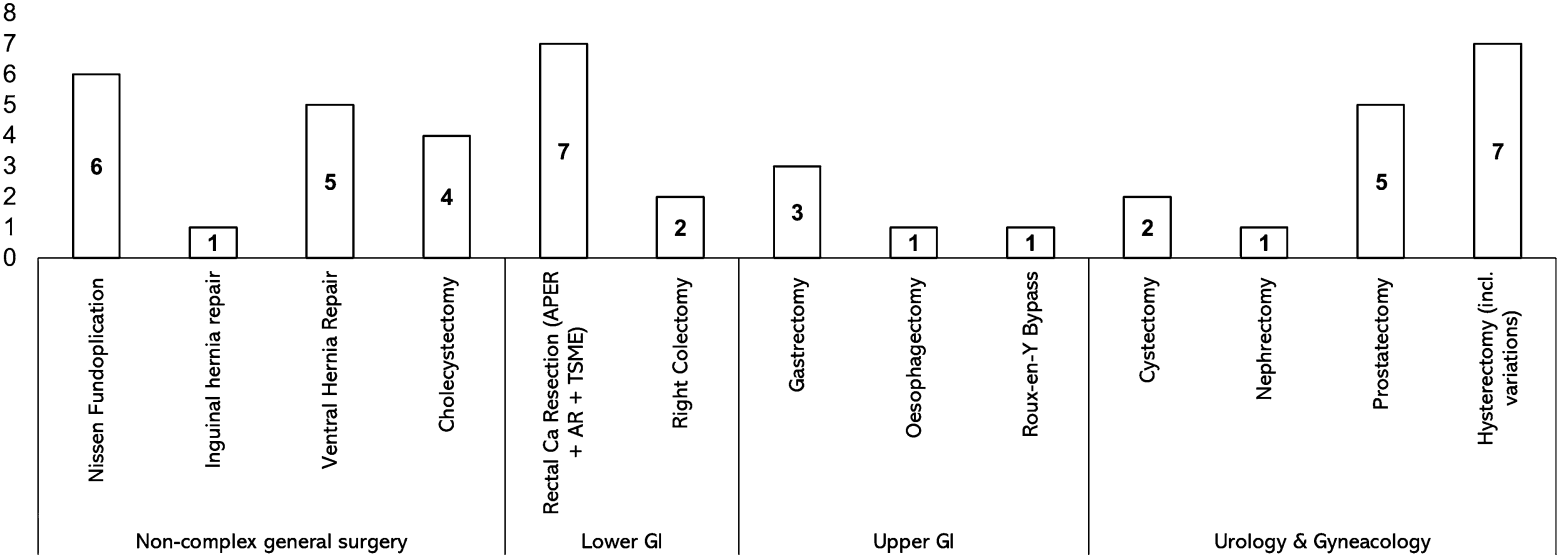
Number of studies per speciality and per procedure

A summary of the literature search can be found in Fig. 1. In total, 45 studies across 13 different surgical procedures were included in the study [15–60]. No studies reported the use of robotic surgery in an emergency setting. All studies reported short-term outcomes, with 25 studies additionally reporting quality of life outcomes, and 6 studies additionally reporting long-term outcomes. No studies were excluded on the basis of reporting only long-term data. Of the 45 studies, 8 pertained to urology, 7 to gynaecology with the remaining 30 studies pertaining to different subspecialties within general surgery (Fig. 2). All of the studies compared robotic surgery with the laparoscopic approach (with one study performing a three-way comparison—open vs laparoscopic vs robotic). Hysterectomy (and its different variations) was the most commonly studied procedure, with all 7 gynaecological RCTs describing this procedure. In all included studies the surgical platform used was the DaVinci robot (Intuitive, California, USA). The main findings of the studies included are summarised in Table S1, Supplementary Digital Content 3.

### Population characteristics

A summary of the population characteristics of the included studies is presented in Table S2, Supplementary Digital Content 4. Overall, 7364 patients were included across all studies (4092 in robotic groups and 3272 in laparoscopic groups), with a 61.4:38.6% male-to-female ratio. The average age in both groups was 57.1 years.

### Short-term outcomes

**Fig. 3:**
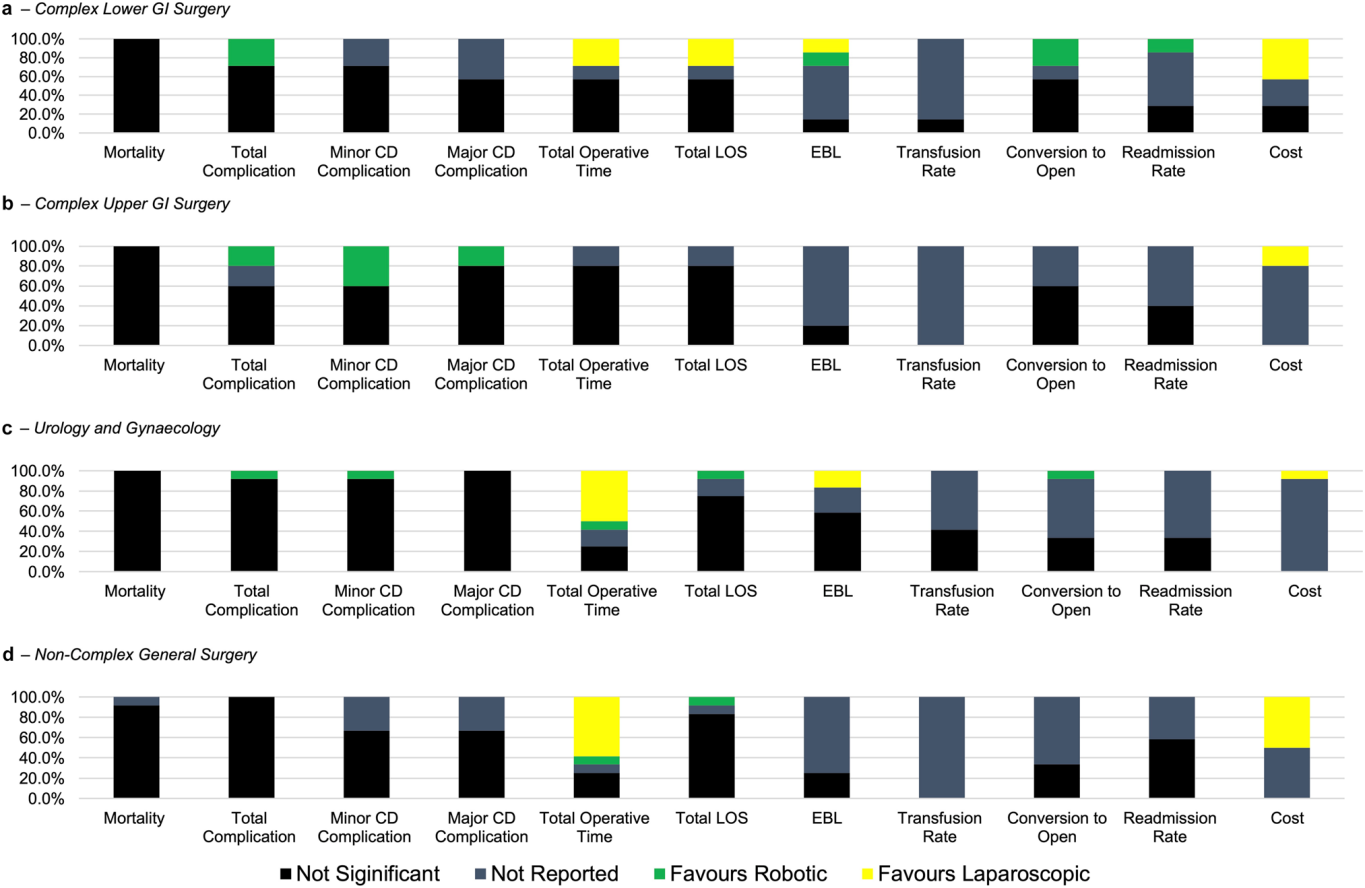
Summary of main intraoperative and short-term post-operative outcomes. Data presented as % of studies either in favour of robotic group, in favour of laparoscopic group (when differences statistically significant *p* < 0.05), not significant or not reported. **a** Complex Lower GI Surgery. **b** Complex Upper GI Surgery. **c** Urology and Gynaecology. **d** Non-complex general surgery

Thirty-six (*n* = 36/45, 80.0%) of the included studies reported intraoperative data, total operative cost, and short-term outcomes. General trends in short-term outcomes are summarised in Fig. 3, with full data available in Table S3, Supplementary Digital Content 5.

None of the studies found a significant difference in 90-day mortality between laparoscopic and robotic groups. Of the 35 studies that included total complication rate, 31 (*n* = 31/35, 88.6%) found no significant difference between the groups, however, 4 studies (*n* = 4/35, 11.4%) found a lower total complication rate in the robotic group. Luo et al. reported a lower complication rate in the robotic hysterectomy (6.7% vs 18.3%, *p* = 0.04) [22], Lu et al. found lower overall morbidity in robotic gastrectomy (9.2% vs. 17.6%, *p* = 0.039) [21], and both trials by Feng et al. showed lower ≥ CD II complication rate for low rectal cancer resection (13.2% vs 23.7%, *p* = 0.013) [51] and low and middle rectal (16.2% vs 23.1%, *p* = 0.003) [50] in the robotic group. No studies found lower complication rates in the laparoscopic groups.

Readmission rates were only reported in 44.4% of included studies (*n* = 16/36), and only one study (*n* = 1/16, 6.3%) found lower readmission rates within 30 days of rectal cancer surgery in the robotic group (2.3% vs 6.9%, *p* = 0.044) [51], with the remaining studies (*n* = 15/16, 93.8%) finding no significant differences.

Total operative time was reported to be significantly longer in the robotic group in 16 (51.6%) out of the 31 studies that reported it. Eleven studies (*n* = 11/31, 35.4%) found no significant differences in operative time. Four studies (*n* = 4/31, 12.9%) (1 in hysterectomy, 1 in Nissen Fundoplication, 1 in oesophagectomy and 1 in Roux-en-Y gastric bypass) have found it to be significantly shorter in the robotic group.

There was no significant difference in estimated blood loss in the majority of studies that reported it (*n* = 12/18, 66.7%), with 3 studies each (*n* = 3/18, 16.7%) reporting in favour of robotic and laparoscopic groups, respectively. No studies found a significant difference in the number of transfusions between the groups.

The conversion rate was reported in 18 studies, and out of those, there was no significant difference in 15 studies (*n* = 15/18, 83.3%). However, 3 studies (*n* = 3/18, 16.7%) found robotic surgery to be associated with lower conversion rates: one in robotic hysterectomy (0% vs 10%, *p* = 0.037) [24], and two in rectal cancer resections (4.0% vs 38.4%, *p* = 0.005 and 0% vs 2.9%, *p* = 0.030) [44, 50].

There was no significant difference in the total postoperative length of stay in 27 studies (*n* = 27/32, 84.4%). Five studies (*n* = 5/32, 16.6%) found the total length of stay to be shorter in the robotic group (two in rectal cancer resection, 1 in gastrectomy, 1 in hysterectomy and 1 in cholecystectomy).

Of the 13 studies that reported total cost, 11 (*n* = 11/13, 84.6%) found it to be significantly higher in the robotic group, with this being found in 1 or more studies of ventral hernia repair, hysterectomy, cholecystectomy, rectal cancer resection, gastrectomy, Nissen fundoplication and right colectomy. Two remaining studies found no significant differences between groups (*n* = 2/13, 15.3%).

### Quality of life data

Twenty-five studies (based on 23 unique RCTs) have included health-related quality of life outcomes or other related patient-reported outcomes such as cosmetic satisfaction*.* A variety of quality of life instruments, including SF-36, pain VAS, FACT-Bi and HRQoL was used. Out of the 23 unique studies, 14 (*n* = 14/23, 60.9%) found no significant difference between robotic and laparoscopic groups, one study (*n* = 1/23, 4.3%) had a significant difference in different metrics favouring either laparoscopic or robotic groups, and 8 favoured robotic groups (*n* = 8/23, 34.8%).

Two studies found that robotic cholecystectomy was associated with significantly higher cosmesis satisfaction and body image (*p* < 0.05 at every follow-up and *p* < 0.001 at 1 month) [20, 36]. Two studies found significantly better sexual function outcomes at 12 months after robotic rectal cancer resection (*p* = 0.03 and *p* < 0.05) [51, 61]. Three studies found robotic prostatectomy to be associated with significantly better QoL outcomes; one found significantly better significantly continence and potency at 12 months (*p* < 0.001), and two found better erectile function (*p* = 0.0002 and *p* = 0.0013) [15, 37, 55]. One study found better hernia-specific QoL after robotic ventral hernia repair at 12 months (92 points vs 77 points, *p* = 0.04) [53].

### Long-term data

Six studies included data on long-term outcomes (one for rectal cancer, one for right colectomy, two for radical cystectomy, one for hysterectomy, and one for radical prostatectomy [17–19, 30, 51, 55]. All of these studies pertained to surgeries performed for malignant diseases. None of the studies found significant differences between robotic and laparoscopic groups in terms of long-term overall survival and disease-free survival outcomes.

### Risk of bias assessment

The summary of the risk of bias assessment is summarised in Figs. S1 and S2, Supplementary Digital Content 6. Overall, 7 (15.6%) studies had a low risk of bias, 30 (66.7%) had some concerns and 8 (17.8%) had a high-risk of bias.

## Discussion

This review identified forty-five randomised clinical trials comparing robotic and laparoscopic surgery across urology, gynaecology and general surgery. There were no significant differences between robotic and laparoscopic surgery in terms of mortality, in all included studies. The majority of studies found no significant differences in complication rates (*n* = 31/35, 88.5%), length of postoperative stay (*n* = 27/32, 84.4%), and lower conversion rates (*n* = 15/18, 83.3%). However, robotic surgery was found to be associated with higher total operative time in 16 (*n* = 16/31, 51.7%) studies, and higher total cost in 11 (*n* = 11/13, 84/6%) studies. In selected studies reporting on health-related quality of life (*n* = 8/23, 34.7%), robotic surgery was associated with better cosmetic outcomes and patient satisfaction, as well as surgery-specific functional outcomes such as erectile function, and continence. Overall, heterogeneity in study populations included, technical approach, and methodologies in randomised trials comparing robotic and laparoscopic surgery complicates the assessment of robotic surgery, across different surgical specialities.

Robotic surgery was shown to be comparable in terms of postoperative complications across all procedure types. Previous studies have commented on the potential advantages of robotic surgery, especially in the context of complex procedures, requiring enhanced precision e.g., gastrectomy or rectal cancer resection [62, 63]. Robotic surgery, due to its endo-wristed instruments, tremor reduction, and 3D vision, can potentially provide higher precision, translating to better outcomes in operations requiring meticulous lymph node dissection, multiple anastomoses, etc [8]. Although it should be interpreted with caution due to the vast majority of trials finding no significant differences, the four studies reporting lower complication rates in robotic (*n* = 4/35, 11.4%), were all pertaining to complex resectional surgery (rectal cancer resection, gastrectomy and hysterectomy). This can be contrasted with trials on non-complex general surgical procedures such as hernia repair or cholecystectomy, where no significant differences were reported.

We observed heterogeneity both in outcome magnitudes and outcome direction i.e., some RCTs favouring the robotic approach, while some favouring laparoscopic with a large variation in morbidity and conversion rates reported within the review. This can be attributed to multiple factors, potentially compounded by the inclusion of procedures from across multiple surgical specialities, and with multiple degrees of complexities, and varied indications (e.g., cholecystectomies and rectal resections).

Moreover, the existence of learning curve effects with new surgical techniques, including robotic surgery, has been shown previously, both in the transition from open to laparoscopic and from laparoscopic to robotic approaches, with the number of procedures needed for proficiency differing across procedures [64]. The learning curve effect was reported for pancreatic surgery (distal pancreatectomy and pancreaticoduodenectomy) [65], prostatectomy [66], and hysterectomy [67], amongst others [64].

It remains an important consideration when comparing laparoscopic and robotic surgery, in light of lack of reporting of surgeon- and unit-experience in the included trials, the effect it might have had on the results in either of the groups are difficult to quantify, highlighting the need of universal reporting of these baseline centre- and surgeon-characteristics in surgical randomised trials. Whether such a learning curve effect is shorter in robotic surgery remains to be seen, however, preliminary data from non-clinical studies suggest a potential for quick skills attainment in novice surgeons, which could have implication for training of future surgeons. [68, 69].

Higher cost and higher operative time were two of the most common aspects in which robotic surgery compared inferiorly to laparoscopic surgery. Robotic surgery has been previously shown to cost on average between $1000 and $4000 more per case than its laparoscopic or endoscopic counterparts [70]. These analyses are largely based on the DaVinci robotic system, which has been used in all RCTs included in this review, and until 2021 one of the only systems on the market. However, new robotic systems, including ones by Medtronic, CMR, and Johnson & Johnson are set to gain a larger share of the market, which could drive the costs down, as competition increases [9]. Nevertheless, currently, in light of comparable complication rates and short-term outcomes, and no healthcare benefits proven, robotic surgery does not provide ‘value for money’ in most cases at present. The expansion of the robotic platform market also has implications for robotic surgery training. Ideally training programmes should adapt their basic simulation training to include all platforms. This would add significantly to the cost of dedicated robotic courses and adds to the complexity of training in robotics. However, mastery of simulation-based curriculum on robotic platforms can be achieved in as little as 4 h, and procedural-specific robotic techniques should not differ significantly between platforms [71]. What is more, as robotics continues to gain momentum in the surgical field, in our opinion should be included in all future residency programmes where possible; it has been shown that through dedicated, protected time during residency, basic robotic surgery competencies can be achieved [71, 72].

While comparable in terms of short-term outcomes, which are commonly used in surgical randomised controlled trials, the potential healthcare benefits of robotic surgery could lie in its impact on patient-reported outcome measures (PROMs). These encompass a variety of metrics including postoperative pain, patient satisfaction, cosmesis and return to function. While 15 out of 23 studies found no significant differences in PROMs, eight studies (*n* = 8/23, 34.7%) found significant improvements across multiple such outcome measures in robotic group. However, only 23 out of 45 studies included (51.1%) incorporated such outcomes into their study design. Going forward, more comprehensive inclusion of PROMs in randomised clinical trials evaluating surgical interventions, as either primary or composite outcomes will be necessary to fully understand if the robotic platform has any inherent advantages to laparoscopic surgery in that domain. Having said that, the benefits of the robotic platform lie in its minimally invasive character, thus potentially limiting its advantage over laparoscopic surgery in this domain.

Another potential area, in which robotic surgery could provide additional value compared to laparoscopic surgery is surgeon fatigue and comfort. Surgeons often report musculoskeletal pain, which is a concern for their longevity and burnout [73, 74]. The robotic platform allows for a more ergonomic surgeon position when compared to laparoscopic surgery, potentially reducing fatigue and pain [75]. Although only two studies included in the review, reported on metrics of surgeon fatigue, these studies highlight the importance of the inclusion of surgeon-oriented outcomes in future trials. The focus, in the studies included in the review, was put on comfort and ergonomics in the short-, rather than long-term, the latter of which is of particular importance [40, 58]. As such, standardised measures of surgical ergonomics such as NASA-TLX, BORG CR-10, or EMG data, collected using questionnaires or wearable sensors should be a part of future randomised trials involving robotic and laparoscopic surgery, to be able to accurately assess and compare robotic and laparoscopic surgery in terms of impact on the surgeons [76, 77].

This review has several limitations. Firstly, the procedure selection, although encompassing surgeries from multiple specialities, limits the applicability of the conclusions. What is more, as only articles in English only were included in the review, selection bias might exist. The decision to limit this review to only grade 1 evidence from randomised controlled trials, while increasing the internal validity of the analysis, resulted in the omission of other evidence from prospective cohort studies, and thus omission of surgical procedures such as major hepatectomies or pancreaticoduodenectomies. Finally, the heterogeneity amongst reporting within RCTs, surgical techniques and populations did not enable meaningful pooled, or meta-analysis, and thus, a qualitative synthesis of evidence was performed instead. However, since trials were most commonly powered for detecting differences in a single, primary endpoint (Table S1), inclusion of data on multiple post-operative outcomes could have increased the risk of type 2 bias in the review, further complicating detection of true differences between the two surgical approaches. Of note, the majority of trials do not have strict criteria for inclusion, or lack reporting of the level surgeon experience in minimally invasive techniques, potentially confounding the results.

This review highlights the shortcomings of the currently published RCTs comparing robotic and laparoscopic surgery. Going forward, more emphasis should be put on incorporating, long-term outcomes (including oncological outcomes for cancer surgery), as well as objective metrics of surgeon performance. When designing RCTs comparing two surgical interventions, the learning curve effect should be considered, to ensure the comparison is devoid of bias. Finally, patient-reported outcome measures should be incorporated into such studies, as patient-centred design can elucidate the true value of robotic surgery.

## Conclusion

Robotic surgery shares many of the advantages of laparoscopic surgery, as they are both minimally invasive approaches. Short-term mortality and morbidity of robotic and laparoscopic surgery are comparable, however, concerns over increased operative time and higher total costs of robotic surgery persist across many common surgical procedures. Currently available randomised controlled trials focus on short-term surgical outcomes, while future studies should also incorporate patient-reported outcomes measures, long-term follow-up, and metrics of surgical fatigue and comfort into their design. As the robotic platform evolves, efforts need to be made to identify procedures in which patient benefit exists, to justify the higher initial cost and longer duration of robotic surgery.

## Supplementary Information

Below is the link to the electronic supplementary material.

Supplementary file1 (DOCX 14 KB)

Supplementary file2 (DOCX 22 KB)

Supplementary file3 (DOCX 27 KB)

Supplementary file4 (DOCX 457 KB)
