# Supplementary material for: Laparoscopic versus robotic abdominal and pelvic surgery: a systematic review of randomised controlled trials
Source: Surg Endosc. 2023 Jul 13;37(9):6672–81. doi: 10.1007/s00464-023-10275-8 (PMC10462573; doi:10.1007/s00464-023-10275-8)
Supplement: Supplementary file 1 — Supplementary file1 (DOCX 14 KB) [file 464_2023_10275_MOESM1_ESM.docx]

**Appendices**

Appendix 1 – Search strategy

1. robotic.mp. or robotics/
2. robot*.ti. or robot*.ab. or robot*.kw.
3. gynecologic surgery/ or general surgery/ or liver surgery/ or rectum surgery/ or spleen surgery/ or laparoendoscopic single site surgery/ or bariatric surgery/ or surgery/ or gastric bypass surgery/ or prostate surgery/ or emergency surgery/ or urethra surgery/ or bypass surgery/ or minimally invasive surgery/ or gastrointestinal surgery/ or male genital system surgery/ or laparoscopic surgery/ or ureter surgery/ or urologic surgery/ or pancreas surgery/ or uterine tube surgery/ or abdominal surgery/ or stomach surgery/ or urinary tract surgery/ or esophagus surgery/ or uterus surgery/ or thyroid surgery/ or colorectal surgery/ or natural orifice transluminal endoscopic surgery/ or bladder surgery/ or open surgery/ or anus surgery/ or intestine surgery/ or biliary tract surgery/ or robot assisted surgery/ or endocrine surgery/ or pelvis surgery/
4. controlled clinical trial/ or randomized controlled trial/
5. randomi?ed.ti. or randomi?ed.ab. or randomi?ed.kw.
6. 1 or 2
7. 4 or 5
8. 3 and 6 and 7

Appendix 2 – List of Included procedures

1. Inguinal hernia repair
2. Ventral hernia repair
3. Total Hysterectomy (incl. radical hysterectomy with bilateral salpingo-oophrectomy)
4. Cholecystectomy
5. Rectal cancer resections (incl. APER, TSME and AR)
6. Right Colectomy
7. Gastrectomy
8. Nissen Fundoplication
9. Oesophagectomy
10. Roux-en-Y bypass
11. Radical cystectomy
12. Nephrectomy
13. Prostatectomy
