# Supplementary material for: Laparoscopic versus robotic abdominal and pelvic surgery: a systematic review of randomised controlled trials
Source: Surg Endosc. 2023 Jul 13;37(9):6672–81. doi: 10.1007/s00464-023-10275-8 (PMC10462573; doi:10.1007/s00464-023-10275-8)
Supplement: Supplementary file 2 — Supplementary file2 (DOCX 22 KB) [file 464_2023_10275_MOESM2_ESM.docx]

Supplementary Table 2 – Demographic characteristics of the included studies.

| **Author** | **Year** | **M in group 1 (%)** | **M in group 2 (%)** | **Age in group 1** | **Age in group 2** | **BMI group 1** | **BMI group 2** | **ASA group 1** | **ASA group 2** | **Previous abdominal surgery (yes %) group 1** | **Previous abdominal surgery (yes %) group 2** |
| --- | --- | --- | --- | --- | --- | --- | --- | --- | --- | --- | --- |
| Prabhu | 2020 | 91.6 | 88.9 | 56.1±14.1 | 57.2± 13.3 | 26.9±4.42 | 24.9±3.24 | NR | NR | NR | NR |
| Petro | 2021 | 59 | 42 | 56 (50 – 70) | 55 (49 – 60) | 35 (31 – 39) | 31 (27 – 36) | 2.89 | 2.5 | NR | NR |
| Petro | 2022 | 59 | 42 | 56 (50 – 70) | 55 (49 – 60) | 35 (31 – 39) | 31 (27 – 36) | 2.89 | 2.5 | NR | NR |
| Olavaria | 2020 | 26 | 37 | 50.1± 13.3 | 48.0±12.9 | NR | NR | NR | NR | 88 | 73 |
| Costa | 2022 | 18 | 19 | 38.9 | 31.5 | 65.2 | 59.7 | 30.5 | 32.6 | NR | NR |
| Dahnani | 2021 | 26 | 37 | 50.1± 13.3 | 48.0±12.9 | NR | NR | NR | NR | 88 | 73 |
| Narducci | 2020 | 0 | 0 | 58 (28 – 85) | 60 (24 – 85) | 25.6 (16.5 – 56.2) | 26.6 (16.7 – 53.8) | NR | NR | 22 | 21 |
| Paraiso | 2013 | 0 | 0 | 43.8 | 45.6 | 29.9 | 31.4 | NR | NR | 80 | 76 |
| Luo | 2018 | 0 | 0 | 65 (62 – 67) | 64 (62 – 66) | NR | NR | NR | NR | 40 | 50 |
| Silva e Silva | 2018 | 0 | 0 | 60 (47 – 69) | 60 (48 – 69) | 31.7 (21.4 – 54.2) | 30.3 (24.8 – 42.7) | NR | NR | NR | NR |
| Deimling | 2016 | 0 | 0 | 42.3±8 | 43.2±8.5 | 30.6±7.8 | 32.1±9.3 | NR | NR | 25 | 34 |
| Sarlos | 2012 | 0 | 0 | 46.3±4.2 | 45.8±6 | 25.7±5 | 26.0±5.3 | NR | NR | 43 | 35 |
| Maenpaa | 2016 | 0 | 0 | 67 ( 43 – 84) | 70 (48 – 83) | 29 (20 – 46) | 29 (20 – 45) | NR | NR | 48 | 47 |
| Selehi | 2017 | 0 | 0 | 66 (39 – 75) | 67 (52 – 75) | 25.6 (16.9 – 38.3) | 26.8 (18.3 – 42.7) | NR | NR | NR | NR |
| Grochola | 2019 | 33.3 | 46.7 | 52.4 (26 – 82) | 51.5 (30 – 78) | 27.3±3.9 | 27.3±4.2 | NR | NR | NR | NR |
| Kudsi | 2017 | 21 | 7 | 45.8±15.5 | 46.5±17.3 | 30.4±6.5 | 31.7±6.7 | 1.98 | 1.94 | NR | NR |
| Pietrabissa | 2015 | NR | NR | NR | NR | NR | NR | NR | NR | NR | NR |
| Zhou | 2006 | 60 | 65 | NR | NR | NR | NR | NR | NR | NR | NR |
| Jayne | 2017 | 67.9 | 67.9 | 64.4±10.98 | 65.5±11.93 | NR | NR | 2.01 | 1.96 | 26.2 | 28.6 |
| Tolstrup | 2018 | 72 | 77 | 63±10.9 | 68±9.9 | 27±4.5 | 28±4.3 | NR | NR | NR | NR |
| Kim | 2018 | 77.3 | 71.2 | 60.4±9.7 | 59.7±11.7 | 24.1±3.3 | 23.6±3.0 | 1.7 | 1.59 | 24.2 | 37 |
| Bolton | 2021 | NR | NR | NR | NR | NR | NR | NR | NR | NR | NR |
| Feng | 2022 | 174 | 173 | 62.1 | 65.3 | 58.2 | 59.5 | NR | NR | 1.31 | 1.36 |
| Feng | 2022 | 586 | 585 | 60.8 | 60.5 | 59.1 | 60.7 | 23.5±3.3 | 23.5±3.1 | 1.5 | 1.5 |
| Park | 2019 | 40 | 45.7 | 62.8±10.5 | 66.5±11.4 | 24.4±2.5 | 23.8±2.7 | 1.69 | 1.45 | NR | NR |
| Park | 2012 | 40 | 45.7 | 62.8±10.5 | 66.5±11.4 | 24.4±2.5 | 23.8±2.7 | 1.69 | 1.45 | 5 (20.0) | 7 (14.7) |
| Baik | 2008 | 77.8 | 77.8 | 57.3±6.3 | 62.0±9.0 | 22.8±1.8 | 24.0±2.5 | 1.33 | 1.55 | 0 | 1 (5.6) |
| Lu | 2021 | 67.7 | 63.60 | 59±10.2 | 59.3±11.3 | 23.2±3.0 | 22.7±3.3 | 1.94 | 1.94 | NR | NR |
| Hua-Feng | 2017 | 63.8 | 73.7 | 65.13±11.81 | 65.67±13.58 | 24.12±1.73 | 23.90±1.63 | 1.22 | 1.22 | NR | NR |
| Ojima | 2021 | 62.4 | 64.7 | 71 (34 – 90) | 72 (40 – 90) | 21.9 (14 – 32.1) | 22.4 (14.0 – 31.9) | 1.7 | 1.65 | 27.4 | 22.7 |
| El Nakadi | 2008 | 66.7 | 72.7 | 44±4 | 48±4 | 25.3±1.2 | 24.8±0.7 | NR | NR | NR | NR |
| Muller-Stich | 2009 | 50 | 60 | 49.6±12.0 | 50.5±12.4 | 29.2±5.8 | 26.2±3.4 | NR | NR | NR | NR |
| Muller-Stich | 2007 | 50 | 60 | 49.6±12.0 | 50.5±12.4 | 29.2±5.8 | 26.2±3.4 | NR | NR | NR | NR |
| Morino | 2006 | 76 | 72 | 43±12.8 | 46.3±11.3 | 25.2±2.9 | 26.1±2.3 | NR | NR | NR | NR |
| Draaisima | 2006 | 64 | 68 | 48.0 (20 – 74) | 52 (27 – 71) | 25.6 (19.1 – 37.2) | 28.7 (19.5 – 46.6) | NR | NR | NR | NR |
| Lang | 2012 | 20 | 20 | 60 | 49.6 | 50.5±12.4 | 29.2±5.8 | 26.2±3.4 | NR | NR | NR |
| Yang | 2022 | 86.2 | 84.7 | 63 (43 – 75) | 63 (42 – 75) | 23.1±2.8 | 23.0 ± 3.1 | NR | NR | NR | NR |
| Sanchez | 2005 | 2 (8.0) | 3 (12.0) | 43.3 | 44.4 | 45.5 | 43.4 | NR | NR | 60 | 50 |
| Khan | 2016 | 85 | 79 | 68.6±6.8 | 68.6±9.9 | 27.5±4.2 | 26.2±4.2 | 1.85 | 2 | NR | NR |
| Khan | 2020 | 85 | 79 | 68.6±6.8 | 68.6±9.9 | 27.5±4.2 | 26.2±4.2 | 1.85 | 2 | NR | NR |
| Würnschimmel | 2014 | 66 | 72 | 62.7±11.1 | 63.9±10.5 | 27.5±5.3 | 28.5±5.4 | NR | NR | NR | NR |
| Porpiglia | 2013 | 100 | 100 | 63.9±6.7 | 64.7±5.9 | 26.2±2.5 | 26.8±2.9 | 2.0±0.5 | 2.1±0.5 | NR | NR |
| Porpiglia | 2016 | 100 | 100 | 63.9±6.7 | 64.7±5.9 | 26.2±2.5 | 26.8±2.9 | 2.0±0.5 | 2.1±0.5 | NR | NR |
| Asimakopoulos | 2011 | 100 | 100 | 59.6±5.4 | 61.1±5.1 | 25.8±2.6 | 26.3±2.2 | NR | NR | NR | NR |
| Stolzenburg | 2022 | 547 | 171 | 100 | 100 | 65 | 65 | 27.2 | 27 | NR | NR |
| Stolzenburg | 2021 | 547 | 171 | 100 | 100 | 65 | 65 | 27.2 | 27 | NR | NR |
