# Supplementary material for: Laparoscopic versus robotic abdominal and pelvic surgery: a systematic review of randomised controlled trials
Source: Surg Endosc. 2023 Jul 13;37(9):6672–81. doi: 10.1007/s00464-023-10275-8 (PMC10462573; doi:10.1007/s00464-023-10275-8)
Supplement: Supplementary file 3 — Supplementary file3 (DOCX 27 KB) [file 464_2023_10275_MOESM3_ESM.docx]

Supplementary Table 3 – Short Term Outcome Data. Data presented as n (%) or median (range) as appropriate.

| Study | 30 day mortality in group 1 (%) | 30 day mortality in group 2 (%) | 90 day mortality in group 1 (%) | 90 day mortality in group 2 (%) | Total complication rate group 1 | Total complication rate group 2 | Minor CD group 1 | Minor CD group 2 | Major CD group 1 | Major CD group 2 | Readmission rate group 1 | Readmission rate group 2 | Total operative time in group 1 (min) | Total operative time in group 2 (min) | EBL group 1 | EBL group 2 | transfusion group 1 | transfusion group 2 | conversion rate group 1 (%) | conversion rate group 2 | LOS group 1 | LOS group 2 | cost group 1 | cost group 2 |
| --- | --- | --- | --- | --- | --- | --- | --- | --- | --- | --- | --- | --- | --- | --- | --- | --- | --- | --- | --- | --- | --- | --- | --- | --- |
| Prabhu 2020 | 0 (0) | 0 (0) | 0 (0) | 0 (0) | 8 (16.7) | 5 (9.3) | 8 (16.7) | 5 (9.3) | 0 (0) | 0 (0) | 4 (8.3) | 2 (3.8) | 75.5 | 40.5 | NR | NR | NR | NR | NR | NR | 5.75 (5–7) | 5.11 (4 –7) | $3258 | $1421 |
| Petro 2021 | 0 (0) | 0 (0) | 0 (0) | 0 (0) | 2 (6.0) | 3 (8.0) | 2 (6.0) | 2 (6.0) | 1 (3.0) | 0 (0) | 1 (3.0) | 1 (3.0 | 146 (123 – 192) | 94 (69 – 116) | NR | NR | NR | NR | 0 (0.0) | 0 (0.0) | 25h (10 – 30h) | 10h (8 – 31) | NR | NR |
| Olavaria 2020 | 0 (0) | 0 (0) | 0 (0) | 0 (0) | 14 (22.0) | 11 (19.0) | 14 (22.0) | 10 (17.0) | 0 (0) | 1 (2.0) | 1 (2.0) | 2 (5.0) | 141±56 | 77±37 | NR | NR | NR | NR | 1 (2.0) | 1 (2.0) | 0 | 0 | $15865 | $12955 |
| Dahmani 2021 | 0 (0) | 0 (0) | 0 (0) | 0 (0) | 14 (22.0) | 11 (19.0) | 14 (22.0) | 10 (17.0) | 0 (0) | 1 (2.0) | 1 (2.0) | 2 (5.0) | 141±56 | 77±37 | NR | NR | NR | NR | 1 (2.0) | 1 (2.0) | 0 | 0 | $15865 | $12955 |
| Narducci 2020 | 0 (0) | 0 (0) | 0 (0) | 0 (0) | 116 (66.0) | 145 (76.0) | 67 (38.0) | 104 (55.0) | 49 (28.0) | 41 (21.0) | NR | NR | 190 (75 – 432) | 145 (33 – 407) | 100 (0 – 2500) | 50 (0 – 1000) | 11 (6.0) | 8 (4.00) | 12 (8.0) | 10 (5.0) | 4.9 | 4.3 | NR | NR |
| Paraiso 2013 | 0 (0) | 0 (0) | 0 (0) | 0 (0) | 0 (0) | 0 (0) | 0 (0) | 0 (0) | 0 (0) | 0 (0) | 0 (0) | 0 (0) | 172±89.0 | 102.7±63.7 | NR | NR | 2 | 1 | NR | NR | 1.4±0.9 | 1.4±0.9 | NR | NR |
| Luo 2018 | 0 (0) | 0 (0) | 1 (3.3) | 2 (6.7) | 4 (6.7) | 11 (18.3) | 4 | 10 | 0 | 1 | NR | NR | NR | NR | NR | NR | NR | NR | NR | NR | 13 (10 – 15) | 15 (11 – 17) | NR | NR |
| Silva e Silva 2018 | 1 | 1 | 0 (0) | 0 (0) | 14 | 10 | 6 | 2 | 9 | 9 | NR | NR | 319 (170 – 520) | 248 (164 – 465) | 162 (0 – 2915) | 105.5 (0 – 1465) | NR | NR | 1 | 2 | 3 (2 – 5) | 3 (2 – 43) | $9655 | $6812 |
| Deimling 2016 | 0 (0) | 0 (0) | 0 (0) | 0 (0) | 3 (4.0) | 6 (8.0) | 3 (4.0) | 6 (8.0) | 0 (0) | 0 (0) | 0 (0) | 0 (0) | 73.9 | 74.9 | NR | NR | NR | NR | NR | NR | 22h (9.5 – 24.0) | 22h (19.0 – 25.0) | NR | NR |
| Sarlos 2012 | 0 (0) | 0 (0) | 0 (0) | 0 (0) | 15 (30.0) | 11 (22.00) | NR | NR | NR | NR | NR | NR | 106±29 | 75±21 | 87±68 | 75±21 | NR | NR | 1 (2.0) | 0 (0) | 3.3±0.9 | 3.6±3.9 | NR | NR |
| Maenpaa 2016 | 0 (0) | 0 (0) | 0 (0) | 0 (0) | 18 (36.0) | 10 (20.0) | 6 (12.0) | 5 (6.0) | 12 (24.0) | 5 (6.0) | NR | NR | 139 (86 – 197) | 170 (126 – 259) | 50 (5 – 500) | 50 (20 – 1200) | 6 (12.0) | 2 (4.0) | 0 (0.0) | 5 (10.0) | 1 (1 – 4) | 2 (1 – 7) | NR | NR |
| Selehi 2017 | 0 (0) | 0 (0) | 0 (0) | 0 (0) | 16 (33.4) | 11 (23.0) | 13 (27.2) | 8 (16.7) | 3 (6.3) | 3 (6.3) | 3 (6.3) | 5 (10.4) | 233 (166 – 320) | 187 (109 – 300) | 78 (20 – 300) | 200 (50 – 850) | 0 (0) | 1 (2.1) | 0 (0.0) | 0 (0.0) | 2 (1 – 5) | 5 (4 – 9) | $18565 | $21505 |
| Grochola 2019 | 0 (0) | 0 (0) | 0 (0) | 0 (0) | 4 (13.3) | 7 (23.3) | 4 (13.3) | 6 (20.0) | 0 (0) | 1 (3.3) | NR | NR | 85.5 (48 – 148) | 74 (31 – 135) | 5.0 (0 – 150) | 3.5 (0 – 300) | NR | NR | 2 (6.7) | 3 (10.0) | 2 (1 – 4) | 2 (1 – 26) | CHF 9734 | CHF 6900 |
| Kudsi 2017 | 0 (0) | 0 (0) | 0 (0) | 0 (0) | 4 (5.0) | 2 (4.0) | NR | NR | NR | NR | NR | NR | 610±27.5 | 44±19.9 | 13.06 | 15.83 | NR | NR | NR | NR | 16.67h | 13.93h | NR | NR |
| Pietrabissa 2015 | 0 (0) | 0 (0) | 0 (0) | 0 (0) | NR | NR | NR | NR | NR | NR | NR | NR | 98±34 | 87±30 | NR | NR | NR | NR | 0 (0) | 0 (0) | 1.2 (1 – 3) | 1.2 (1 – 3) | NR | NR |
| Zhou 2006 | NR | NR | NR | NR | 0 (0) | 0 (0) | 0 (0) | 0 (0) | 0 (0) | 0 (0) | 0 (0) | 0 (0) | 104±20.5 | 78±17.1 | 36.5±25.2 | 31.8±16.4 | NR | NR | NR | NR | 2.8±0.8 | 2.8±0.7 | NR | NR |
| Jayne 2017 | 2 (0.8) | 2 (0.9) | NR | NR | 78 (33.1) | 73 (31.7) | NR | NR | NR | NR | NR | NR | 298.5±88.71 | 261.0±83.24 | NR | NR | NR | NR | 19 (8.1) | 28 (12.2) | 8.0±5.85 | NR | $13668 | $12556 |
| Tolstrup 2018 | 1 (4.0) | 0 (0) | NR | NR | 9 (36.0) | 10 (38.4) | 7 (28.0) | 5 (19.2) | 2 (8.0) | 5 (19.2) | NR | NR | 152±43 | 170±57 | NR | NR | NR | NR | 1 (4.0) | 10 (40.0) | 8.9±5.6 | 9.5±7.7 | NR | NR |
| Kim 2018 | 0 (0) | 0 (0) | 0 (0) | 0 (0) | 23 (34.8) | 17 (23.3) | 17 (25.8) | 13 (17.8) | 6 (9.4) | 4 (5.4) | NR | NR | 339.2±80.1 | 227.8±65.6 | 100 (0 – 1000) | 50 (0 – 300) | NR | NR | 1 (1.5) | 0 (0) | 10.3±3.4 | 10.8±7.4 | NR | NR |
| Bolton 2021 | 2 (0.8) | 2 (0.9) | NR | NR | 78 (33.1) | 73 (31.7) | NR | NR | NR | NR | NR | NR | 298.5±88.71 | 261.0±83.24 | NR | NR | NR | NR | 19 (8.1) | 28 (12.2) | 8.0±5.85 | NR | $13668 | $12556 |
| Park 2019 | 0 (0) | 0 (0) | 0 (0) | 0 (0) | 6 (14.7) | 7 (20.0) | NR | NR | NR | NR | 1 (2.8) | 2 (5.6) | 195±41 | 129±43.2 | 35.8±36.3 | 46.8±31.3 | NR | NR | 0 (0) | 0 (0) | 7.9±4.1 | 8.3±4.2 | $12235 | $10320 |
| Park 2012 | 0 (0) | 0 (0) | 0 (0) | 0 (0) | 6 (14.7) | 7 (20.0) | 5 (11.9) | 6 (14.7) | 1 (2.8) | 1 (2.8) | 1 (2.8) | 2 (5.6) | 195±41 | 129±43.2 | 35.8±36.3 | 46.8±31.3 | NR | NR | 0 (0) | 0 (0) | 7.9±4.1 | 8.3±4.2 | $12235 | $10320 |
| Baik 2008 | 0 (0) | 0 (0) | 0 (0) | 0 (0) | 3 (12.5) | 2 (8.33) | 3 (12.5) | 2 (8.33) | 0 (0) | 0 (0) | NR | NR | 217.1±51.6 | 204.3±51.9 | NR | NR | 1 (5.5) | 1 (5.5) | 0 (0) | 2 (11.1) | 6.9±1.3 | 8.7±1.3 | NR | NR |
| Lu 2021 | 0 (0) | 0 (0) | 0 (0) | 0 (0) | 13 (9.2) | 25 (17.6) | 11 (7.8) | 22 (15.5) | 2 (1.4) | 3 (2.1) | 2 (1.4) | 2 (1.4) | NR | NR | NR | NR | NR | NR | NR | NR | 7.9±4.3 | 8.2±2.5 | NR | NR |
| Hua-Feng 2017 | 0 (0) | 0 (0) | 0 (0) | 0 (0) | 5 (5.0) | 12 (19.7) | 4 (4.0) | 9 (14.8) | 1 (1.0) | 3 (4.9) | NR | NR | 153.11±16.44 | 151.97±23.58 | 41.27±20.23 | 83.69±32.81 | NR | NR | NR | NR | 3.75±0.74 | 5.36±1.24 | NR | NR |
| Ojima 2021 | 0 (0) | 0 (0) | 0 (0) | 0 (0) | NR | NR | 10 (8.5) | 23 (19.3) | 6 (5.3) | 19 (16.2) | NR | NR | 297 (170 – 654) | 245 (131 – 534) | 25 (5 – 475) | 25 (5 – 1405) | NR | NR | NR | NR | 12 (7 – 43) | 13 (6 – 45) | NR | NR |
| El Nakadi 2008 | 0 (0) | 0 (0) | 0 (0) | 0 (0) | 4 (36.3) | 3 (33.3) | NR | NR | NR | NR | NR | NR | 137±12 | 96±5 | NR | NR | NR | NR | NR | NR | 4.4±0.2 | 4.1±0.3 | NR | NR |
| Muller-Stich 2009 | NR | NR | NR | NR | NR | NR | NR | NR | NR | NR | NR | NR | NR | NR | NR | NR | NR | NR | NR | NR | NR | NR | NR | NR |
| Muller-Stich2007 | 0 (0) | 0 (0) | 2 (0) | 3 (0) | 16 (80.0) | 18 (90.0) | 16 (80.0) | 18 (90.0) | 0 (0) | 0 (0) | 0 (0) | 0 (0) | 88±18 | 102±19 | 0 (0) | 0 (0) | NR | NR | NR | NR | 2.9±0.8 | 3.3±0.9 | € 3,244 | € 2,743 |
| Morino 2006 | 0 (0) | 0 (0) | 2 (0) | 3 (0) | 4 (0) | 5 (0) | 6 (0) | 7 (0) | 8 (0) | 9 (0) | 10 (0) | 11 (0) | 131.3±18.3 | 91.1±10.6 | NR | NR | NR | NR | NR | NR | 2.9 (2 – 6) | 3.0 (2 – 7) | € 3,157 | € 1,527 |
| Draaisima 2006 | 0 (0) | 0 (0) | 0 (0) | 0 (0) | 0 (0) | 3 (12.0) | 0 (0) | 3 (12.0) | 0 (0) | 0 (0) | NR | NR | 120 (80 – 180) | 95 (60 – 210) | 20 (0 – 200) | 45 (0 – 200) | NR | NR | NR | NR | 3 (2 – 6) | 3 (1 – 13) | NR | NR |
| Yang 2022 | 0 (0) | 1 (0.6) | 1 (0.6) | 1 (0.6) | 88 (48.6) | 74 (41.8) | 66 (36.4) | 56 (26.2) | 22 (12.2) | 18 (10.2) | NR | NR | 203.8 ± 59.4 | 244.9 ± 61.0 | 200 (100 – 400) | 200 (100 – 500) | NR | NR | 7 (3.9) | 6 (3.4) | NR | NR | NR | NR |
| Sanchez2005 | 0 (0) | 0 (0) | 0 (0) | 0 (0) | 0 (0) | 0 (0) | 0 (0) | 0 (0) | 0 (0) | 0 (0) | NR | NR | 130.8 | 149.4 | NR | NR | NR | NR | NR | NR | NR | NR | NR | NR |
| Khan 2016 | 0 (0) | 0 (0) | 0 (0) | 0 (0) | 11 (55) | 5 (26.0) | 5 (25.0) | 4 (21.0) | 6 (30.0) | 1 (5.0) | NR | NR | 389±98 | 301±51 | 585±618 | 460±485 | NR | NR | NR | NR | 11.9±6.2 | 9.7±3.6 | NR | NR |
| Khan 2020 | 0 (0) | 0 (0) | 0 (0) | 0 (0) | 11 (55) | 5 (26.0) | 5 (25.0) | 4 (21.0) | 6 (30.0) | 1 (5.0) | NR | NR | 389±98 | 301±51 | 585±618 | 460±485 | NR | NR | NR | NR | 11.9±6.2 | 9.7±3.6 | NR | NR |
| Würnschimmel 2014 | 0 (0) | 0 (0) | 0 (0) | 0 (0) | 14(21.0) | 18 (31.0) | 6 | 14 | 8 | 4 | NR | NR | 230.2±5.92 | 192.3±44.5 | NR | NR | NR | NR | NR | NR | 6.1±2.9 | 6.3±3.8 | NR | NR |
| Porpiglia 2013 | 0 (0) | 0 (0) | 0 (0) | 0 (0) | 9 (15.0) | 6 (10.0) | 9 (15.0) | 6 (10.0) | 0 (0) | 0 (0) | NR | NR | 147.6±27.1 | 138.1±29.7 | 202.0±124.0 | 234.1±150.1 | NR | NR | NR | NR | 4.6±2.1 | 4.8±1.9 | NR | NR |
| Porpiglia 2016 | 0 (0) | 0 (0) | 0 (0) | 0 (0) | 9 (15.0) | 6 (10.0) | 9 (15.0) | 6 (10.0) | 0 (0) | 0 (0) | NR | NR | 147.6±27.1 | 138.1±29.7 | 202.0±124.0 | 234.1±150.1 | NR | NR | NR | NR | 4.6±2.1 | 4.8±1.9 | NR | NR |
| Asimakopoulos 2011 | 0 (0) | 0 (0) | 0 (0) | 0 (0) | 8 (15.0) | 5 (8.7) | 7 (13.4) | 5 (8.7) | 1 (1.9) | 0 (0) | NR | NR | NR | NR | NR | NR | 0 (0) | 3 (5.0) | 0 (0.0) | 0 (0.0) | NR | NR | NR | NR |
