# Supplementary material for: Laparoscopic versus robotic abdominal and pelvic surgery: a systematic review of randomised controlled trials
Source: Surg Endosc. 2023 Jul 13;37(9):6672–81. doi: 10.1007/s00464-023-10275-8 (PMC10462573; doi:10.1007/s00464-023-10275-8)

*Supplementary Figure 1 – Risk of Bias Assessment using ROB-2 tool; breakdown of each domain in terms of low risk, some concern and high risk (%).*

*Supplementary Figure 2 – Risk of Bias Assessment using ROB-2 tool; breakdown of all the domain scores for all studies include*


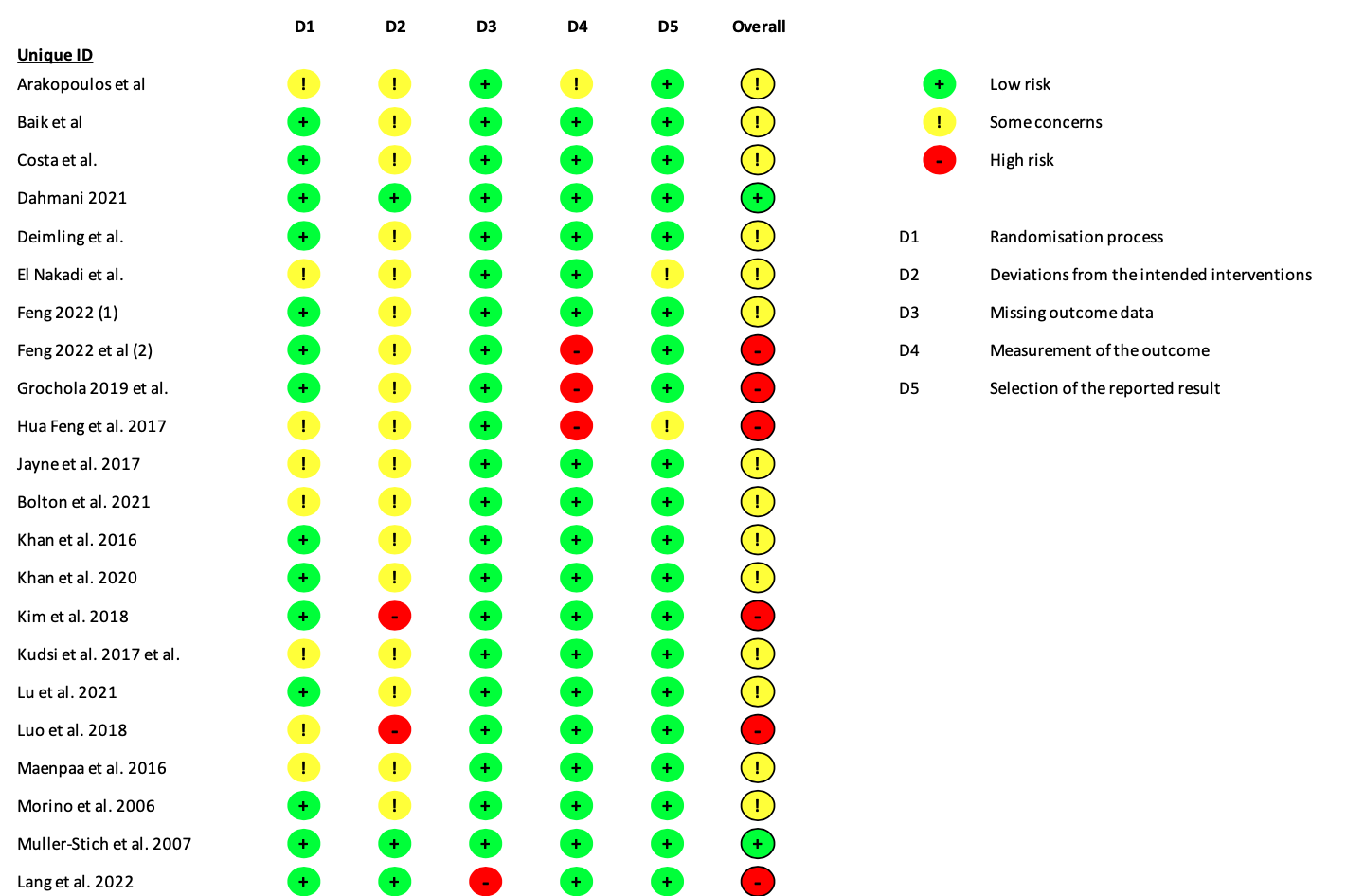


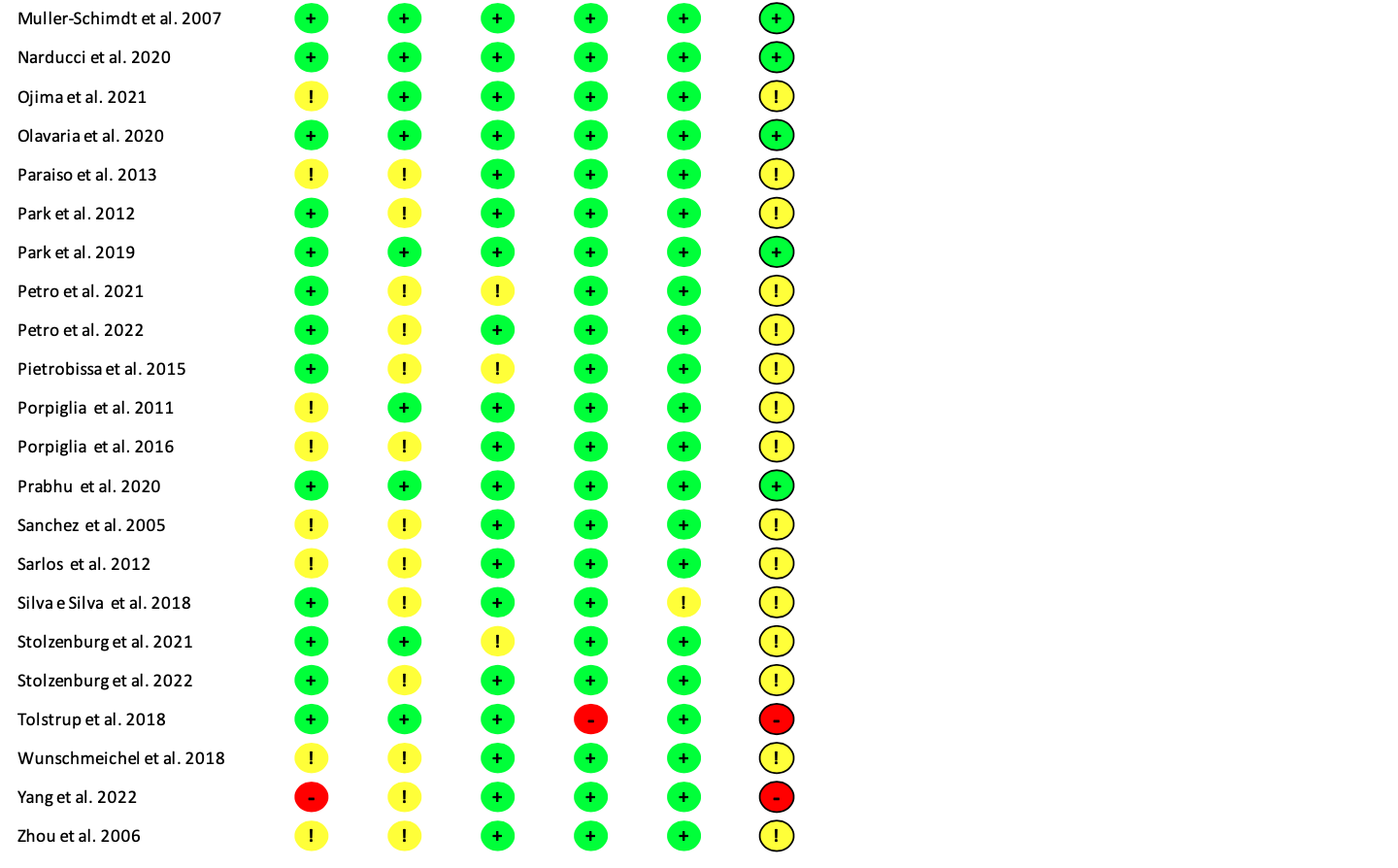

Supplement: Supplementary file 4 — Supplementary file4 (DOCX 457 KB) [file 464_2023_10275_MOESM4_ESM.docx]
